# Roles of tumor-associated macrophages in triple-negative breast cancer progression

**DOI:** 10.3389/fimmu.2025.1677363

**Published:** 2025-10-09

**Authors:** Tianhai Wu, Yuling Liao

**Affiliations:** ^1^ Guangdong Medical University, Zhanjiang, Guangdong, China; ^2^ Department of Breast Surgery, Huizhou First Hospital, Guangdong Medical University, Huizhou, Guangdong, China

**Keywords:** triple-negative breast cancer, tumor-associated macrophages, tumor microenvironment, immune evasion, TAM

## Abstract

Triple-negative breast cancer (TNBC) is an aggressive subtype characterized by the absence of estrogen receptor (ER), progesterone receptor (PR), and human epidermal growth factor receptor 2 (HER2) expression. It is associated with a high risk of recurrence, metastasis, and limited therapeutic options. Tumor-associated macrophages (TAMs) play a central role in TNBC progression by shaping an immunosuppressive tumor microenvironment. Primarily polarized toward an M2-like phenotype under the influence of cytokines such as IL-10 and TGF-β, TAMs facilitate tumor growth, angiogenesis, metastasis, and immune evasion through multiple mechanisms. This review summarizes current understanding of TAM recruitment, polarization, and pro-tumoral functions in TNBC, and outlines emerging therapeutic strategies aimed at depleting TAMs, reprogramming them to an anti-tumor M1-like state, or blocking the CD47-SIRPα phagocytosis checkpoint. These approaches offer promising avenues for reprogramming the TNBC microenvironment and improving clinical outcomes.

## 1 Introduction

Triple-negative breast cancer (TNBC) is a clinically aggressive subtype marked by a high propensity for recurrence and distant metastasis (1, 2). Despite standard regimens, many patients rapidly relapse (3, 4). Recent advances have shifted the focus from tumor-intrinsic traits to the tumor microenvironment (TME), a dynamic niche that orchestrates cancer initiation and progression in concert with tumor cells (5–9). Among TME components, tumor-associated macrophages (TAMs)—the most prevalent innate immune cells—can constitute up to 71.4% of the immune infiltrate in TNBC, far surpassing that in other malignancies (10).

TAMs are implicated across the entire course of TNBC development, from early tumorigenesis to metastatic dissemination, and correlate with poor clinical outcomes (11–13). In TNBC, TAMs adopt either pro-inflammatory M1 or immunosuppressive M2 phenotypes in response to cytokine cues (14). M1 macrophages enhance anti-tumor immunity via inflammatory mediator release, antigen presentation, and tumor cell phagocytosis, whereas M2 macrophages facilitate tumor progression by promoting proliferation, angiogenesis, immune evasion, and metastatic potential (15, 16). During tumor progression, TAMs predominantly exhibit an M2-like phenotype, thereby facilitating TNBC initiation and advancement (17). This review provides a comprehensive overview of the ontogeny, recruitment mechanisms, and polarization dynamics of TAMs in the TNBC microenvironment, and delineates their multifaceted roles in disease progression. In addition, we summarize recent advances in TAM-targeted therapeutic strategies aimed at improving outcomes in TNBC patients.

## 2 Origin, recruitment, and polarization of TAMs

### 2.1 Origin and recruitment of TAMs

Current evidence indicates that TAMs originate from two principal sources. The first comprises bone marrow–derived myeloid progenitors that differentiate into circulating monocytes, which infiltrate the TME and mature into macrophages (18). The second involves embryonic progenitors from the yolk sac or fetal liver that give rise to tissue-resident macrophages (TRMs), which are seeded into organs during development and sustained independently of hematopoietic input through local proliferation (19). Notably, both embryonically derived and monocyte-derived TAMs have been documented in several malignancies, including breast cancer (20). The recruitment of TAMs into the TME of TNBC is primarily mediated by tumor-secreted growth factors and chemokines. Colony-stimulating factor 1 (CSF-1), through binding to its receptor CSF-1R, plays a pivotal role in the recruitment and differentiation of peripheral blood monocytes into TAMs (21). TNBC cells produce substantially higher levels of CSF-1 than non-TNBC subtypes, promoting robust TAM infiltration (22, 23). In parallel, CC chemokine ligand 2 (CCL2) drives monocyte chemotaxis via CCR2 signaling (24), while CCL5 contributes to TAM aggregation and enhances tumor invasion. Importantly, CCL5-CCR3 signaling in tumor cells correlates with poor prognosis in TNBC (25). Immunohistochemical analyses of tumors from 40 TNBC patients further demonstrate that CCL5 production by peritumoral adipose tissue potentiates invasion and metastasis (26). Moreover, VEGF has also been implicated in TAM recruitment, with elevated VEGF levels in TNBC strongly associated with increased macrophage infiltration (27).

### 2.2 Polarization of TAMs

Upon recruitment into the TME, TAMs acquire distinct functional phenotypes shaped by local cues (28). Exposure to lipopolysaccharide (LPS) and IFN-γ drives macrophages toward a classically activated, pro-inflammatory M1-like state, whereas anti-inflammatory cytokines such as IL-10 and TGF-β promote an alternatively activated, immunosuppressive M2-like phenotype (29). M1-like TAMs exert antitumor functions via the production of reactive oxygen species (ROS), nitrogen intermediates, and enhaantigen presentation to T cellnced s (30). By contrast, M2-like TAMs facilitate tumor progression by mediating tissue remodeling, angiogenesis, and immune suppression (28). The TME promotes a phenotypic shift from M1 to M2 polarization through sustained exposure to IL-10, TGF-β, and other tumor-derived factors (31). Additionally, interactions with the extracellular matrix and neoplastic signals further reinforce M2-skewed polarization in TAMs, particularly in TNBC, where such phenotypes dominate the immune landscape (32). This biased polarization underpins the immunosuppressive and pro-tumoral roles of TAMs in TNBC progression. Besides, cytokines activate downstream intracellular signaling pathways that orchestrate M2 polarization. IL-10 predominantly signals through the JAK1/STAT3 axis, where phosphorylated STAT3 translocates to the nucleus and induces the expression of M2-associated genes such as IL-10 and arginase-1 (33, 34). Similarly, IL-4 and IL-13 activate the STAT6 pathway, which promotes transcription of M2 markers including CD206 (35, 36). In addition, TGF-β signaling induces M2 polarization through activation of the PI3K/Akt and SMAD pathways, enhancing the expression of anti-inflammatory and pro-tumoral mediators (37, 38). Notably, activation of the PI3K/Akt axis has also been implicated in metabolic reprogramming of TAMs toward an oxidative phosphorylation (OXPHOS)-dominant state, further supporting their M2-like phenotype and immunosuppressive functions (39, 40).

TNBC progression has been modeled by co-injecting RAW264.7 macrophages and 4T1 TNBC cells into murine mammary ducts (40). During the transition from *in situ* carcinoma to invasive statue, this co-injection approach resulted in suppressed expression of the M1-associated cytokine IL-12 and elevated levels of the M2-associated cytokine TGF-β1 (41). These immunological alterations were accompanied by both lymphatic and pulmonary metastases (42). Additionally, increased concentrations of MMP-8 and VEGF were detected in peripheral blood—both recognized modulators of macrophage polarization (43). These findings suggest that tumor-induced M2 polarization of TAMs may operate through a reinforcing positive feedback loop. MicroRNAs (miRNAs), a class of non-coding single-stranded RNAs with dual oncogenic and tumor-suppressive roles, have emerged as key regulators of TAM polarization (44, 45). For instance, co-culturing miR-200c–overexpressing MDA-MB-231 TNBC cells with RAW264.7 macrophages enhanced expression of M2 markers such as CD206 and IL-10, indicating a role for miR-200c in promoting M2-like phenotypes (46). Conversely, miR-34a has been implicated in facilitating M1 polarization. Using viral transduction to manipulate miR-34a expression in MDA-MB-231 cells followed by co-culture with THP-1 monocytes, it was observed that tumor cells expressing miR-34a more effectively induced M1 polarization compared to miR-34a–silenced controls (17). In addition to miR-200c and miR-34a, other miRNAs such as miR-21 and miR-155 have also been implicated in TAM regulation within the breast cancer microenvironment. miR-21, commonly upregulated in breast cancer, promotes M2 polarization by targeting PTEN and enhancing PI3K/Akt signaling, thereby reinforcing the immunosuppressive phenotype of TAMs (47, 48). Conversely, miR-155 facilitates M1 polarization by inhibiting suppressor of cytokine signaling 1 (SOCS1), leading to enhanced pro-inflammatory cytokine production and tumoricidal activity (49, 50).

## 3 The role of TAMs in TNBC progression

### 3.1 Promotion of tumor cell proliferation

**Figure 1:**
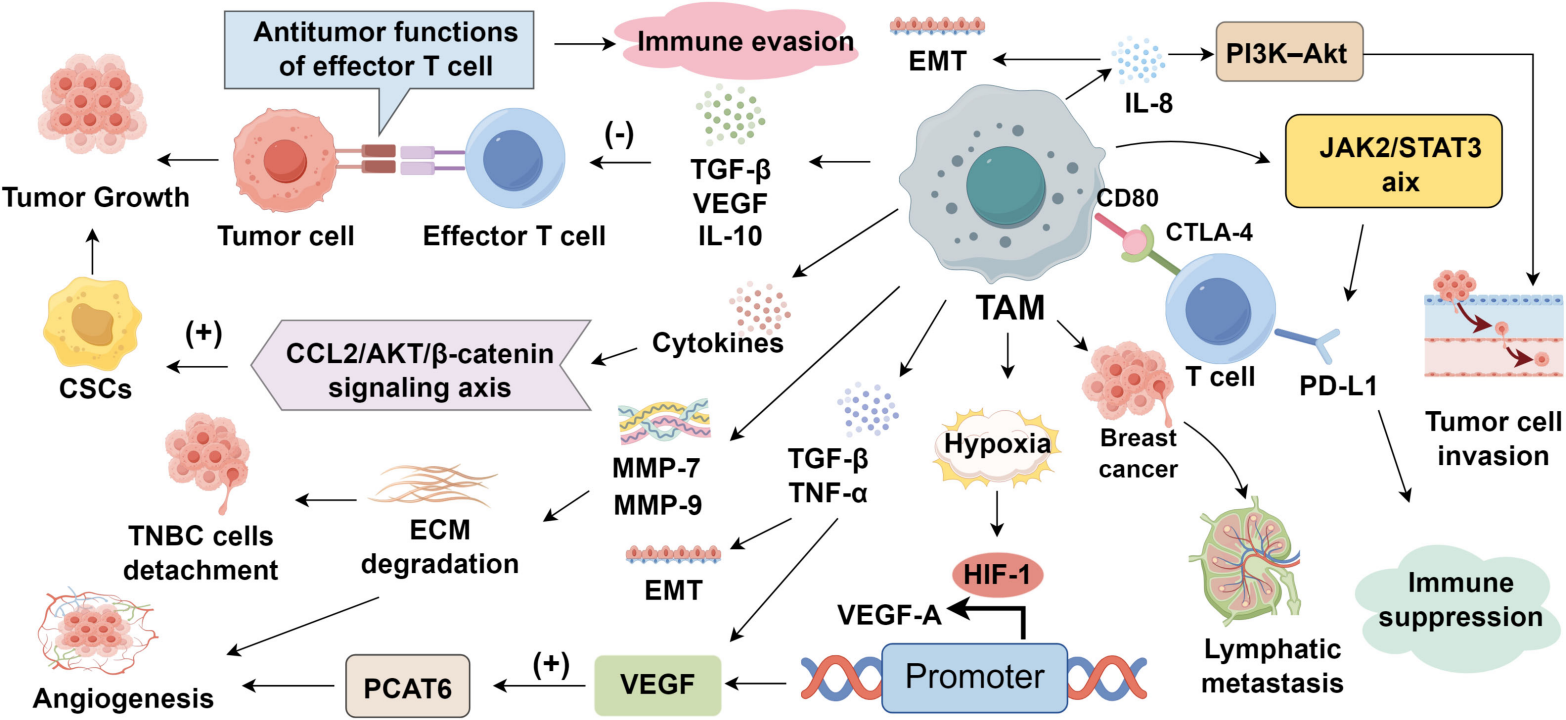
Roles of tumor-associated macrophages in triple-negative breast cancer progression.

The infiltration of TAMs in the TNBC microenvironment is tightly associated with enhanced tumor cell proliferation (51). TAMs secrete various signaling molecules, including TGF-β, VEGF, and IL-10, which suppress the antitumor functions of effector T cells, thereby facilitating tumor cell growth (52, 53). Notably, TAMs also support the maintenance and expansion of cancer stem cells (CSCs), a subpopulation endowed with self-renewal and tumor-initiating capacity, through a variety of paracrine signaling pathways (54, 55). For instance, TAM-derived IL-6 activates STAT3 signaling in TNBC cells, reinforcing stem-like traits and contributing to chemoresistance (56). Similarly, IL-8 promotes the CSC phenotype by upregulating ALDH1, while concurrently activating PI3K/AKT/mTOR and NF-κB signaling cascades (57–59). Moreover, cytokine-driven activation of the CCL2/AKT/β-catenin axis by TAMs further potentiates CSC maintenance and tumor aggressiveness, ultimately fostering TNBC progression and resistance to therapy (60) (**Figure 1**).

### 3.2 Induction of angiogenesis and lymphangiogenesis

The vascular network plays a pivotal role in sustaining tumor growth by delivering oxygen and nutrients, while also serving as a conduit for metastatic dissemination (61). In TNBC, TAMs drive angiogenesis via several key mechanisms (62). First, TAMs secrete matrix metalloproteinases (MMPs), notably MMP-7 and MMP-9, along with cathepsins, to degrade the extracellular matrix (ECM), thereby facilitating endothelial cell invasion and neovessel formation (63). Second, the hypoxic tumor microenvironment polarizes macrophages toward a pro-tumoral TAM phenotype. These cells accumulate in hypoxic niches and upregulate hypoxia-inducible factor-1 (HIF-1), which transcriptionally activates proangiogenic genes such as VEGF-A (64). Third, TAMs amplify VEGF expression through the secretion of TGF-β and TNF-α, further enhancing neovascularization (65). Recent studies have shown that TAM-derived VEGF promotes angiogenesis in TNBC by upregulating prostate cancer-associated transcript 6 (PCAT6) (66). In addition to angiogenesis, TAMs promote lymphangiogenesis, a process critical for lymphatic metastasis (67). Tumor-induced expression of integrin β4 in macrophages enhances their chemotactic aggregation and adhesion to lymphatic vessels, where they secrete TNF-β1, triggering lymphatic endothelial contraction. These macrophages also elevate vascular permeability and disrupt perivascular structures, collectively fostering lymphatic remodeling and tumor cell dissemination through lymphatic routes (68).

### 3.3 Facilitation of metastasis

Metastasis remains the leading cause of death in patients with TNBC, with TAMs playing a pivotal role in promoting tumor cell invasion and dissemination (69). Through secretion of MMPs, TAMs degrade and remodel the ECM, thereby facilitating detachment of tumor cells from the primary site (70). A hallmark of this invasive transition is epithelial-to-mesenchymal transition (EMT), wherein epithelial cells acquire mesenchymal properties and heightened motility (71). TAMs induce EMT via cytokine secretion, including TGF-β (38), TNF-α (12), and IL-4 (72). TGF-β, in particular, triggers EMT-associated transcriptional programs by engaging tumor cell receptors and activating downstream pathways (73). In TNBC, IL-8 further promotes EMT and invasiveness through PI3K–Akt signaling (74). Beyond soluble mediators, TAM-derived exosomes are potent effectors of EMT and metastatic reprogramming (75). Notably, M2-like TAMs release exosomes enriched in miR-223, which activates β-catenin signaling and suppresses epithelial markers such as E-cadherin, thereby enhancing cellular plasticity and invasive capacity (76, 77). TAM-derived exosomal MMPs, particularly MMP-9, degrade ECM components and compromise basement membrane integrity (78, 79). Within the pre-metastatic niche (PMN), these exosomes further contribute to stromal cell recruitment, vascular leakage, and the establishment of a supportive microenvironment for metastatic colonization (80).

### 3.4 Induction of immunosuppression and immune evasion

Immunosuppression is a fundamental prerequisite for tumor initiation and sustained progression (81–83). In TNBC, TAMs exert profound immunoregulatory functions that suppress anti-tumor immunity (84). One key mechanism involves the expression of CD80 and CD86 on TAMs, which engage cytotoxic T-lymphocyte-associated protein 4 (CTLA-4) on T cells to block activation and cell cycle progression, thereby promoting T cell anergy (85). Additionally, TAMs secrete immunosuppressive cytokines such as TGF-β and IL-10 within the TNBC microenvironment, directly impairing the cytotoxic capacity of effector T cells and facilitating immune evasion (86). A central axis in TAM-mediated immunosuppression is the PD-1/PD-L1 pathway. PD-1 ligation by PD-L1 inhibits T cell effector function, and TNBC tissues show markedly elevated PD-L1 expression compared to other subtypes, contributing to enhanced T cell suppression (84). Notably, TNBC cells induce TAMs to upregulate PD-L1 via the JAK2/STAT3 pathway, which further inhibits CD8^+^ T cell–mediated cytotoxicity (87). Moreover, PD-1 is also expressed on TAMs themselves, particularly those with an M2-like phenotype. High PD-1 expression on TAMs is associated with diminished phagocytosis and impaired anti-tumor responses. Blocking PD-1/PD-L1 signaling not only restores macrophage function but also suppresses tumor growth and prolongs survival in murine models (88). In addition to PD-1/PD-L1, other immune checkpoints such as T cell immunoglobulin and mucin-domain containing-3 (TIM-3), lymphocyte-activation gene 3 (LAG-3), and V-domain Ig suppressor of T cell activation (VISTA) are increasingly recognized in TNBC immunotherapy (89–91). These molecules are also regulated by TAMs and contribute to the suppression of T cell activity and immune evasion. For example, TIM-3 and LAG-3 are frequently co-expressed with PD-1 on exhausted T cells in the TNBC microenvironment, and their ligands, including galectin-9 and MHC class II, can be upregulated by TAMs (92–94). VISTA, predominantly expressed on myeloid cells such as TAMs, mediates immune suppression by dampening T cell proliferation and cytokine production (95, 96). Collectively, these findings highlight the role of TAMs as critical regulators of immunosuppression and immune escape in TNBC. Targeting these alternative checkpoints alongside PD-1/PD-L1 may offer synergistic immunotherapeutic benefits in TNBC.

## 4 Therapeutic strategies targeting TAMs

### 4.1 Depleting TAM populations

Given the critical role of TAMs in orchestrating immunosuppressive TME and promoting cancer progression, substantial preclinical and clinical efforts have focused on TAM-targeted interventions (13, 97). These strategies fall into three principal categories: depletion of TAMs, reprogramming toward anti-tumor phenotypes, and blockade of the CD47–SIRPα axis (98–100). Colony-stimulating factor 1 (CSF-1) facilitates the recruitment of TAMs into the TME of breast cancer, where they promote tumor invasion and metastasis. CSF-1 binds its receptor CSF-1R to regulate macrophage survival and trafficking. Pharmacological inhibition of CSF-1R not only reduces TAM infiltration but also delays tumor growth and dissemination (101). Emactuzumab (RG7155), a monoclonal antibody targeting CSF-1R, depletes TAMs by blocking receptor activation (102). Preclinical studies revealed that RG7155 markedly suppressed TAM levels and enhanced T cell infiltration (103). However, a Phase I clinical trial evaluating RG7155 in breast cancer demonstrated no significant clinical benefit when administered alone or in combination with paclitaxel, despite successful TAM suppression (104). These findings highlight the necessity of thoroughly evaluating the TME before initiating CSF-1R-targeted therapies.

Chemokines also critically regulate TAM recruitment (105). CCL2 recruits circulating monocytes that differentiate into TAMs via its receptor CCR2 (106). Inhibition of CCL2 has been shown to attenuate TAM infiltration and impair cancer stem cell renewal, thereby restraining TNBC progression (60, 107). However, abrupt withdrawal of CCL2 blockade can trigger a rebound effect, marked by increased TAM accumulation, enhanced metastasis, and reduced survival in preclinical breast cancer models (108). This underscores the limitations of monotherapy targeting CCL2 in metastatic disease and emphasizes the importance of understanding TME composition and antitumor dynamics. CCL5 is a key modulator of tumor growth and metastatic dissemination, and its receptor CCR5 is frequently overexpressed in TNBC (109). Elevated CCL5 levels correlate with increased tumor burden following neoadjuvant chemotherapy (110), and gene expression profiling of residual tumors reveals enrichment of CCL5, suggesting its role in recruiting macrophages and fostering recurrence (111). Thus, targeting the CCL5/CCR5 axis emerges as a promising approach to limit TAM-driven relapse in TNBC.

### 4.2 Reprogramming TAMs toward an anti-tumor phenotype

Macrophages exhibit remarkable plasticity and can dynamically shift their phenotype in response to environmental cues. Reprogramming TAMs from a tumor-promoting (M2-like) to an inflammatory, tumoricidal (M1-like) phenotype offers a promising avenue for TNBC therapy (112, 113). CD40, a member of the TNF receptor superfamily, is expressed on antigen-presenting cells, including macrophages and B cells (114). Engagement of CD40 by CD40L triggers the production of TNF, ROS, and nitric oxide (NO), and promotes T cell activation and antitumor immunity (115). In preclinical studies, CD40 agonists have successfully reprogrammed TAMs into M1-like macrophages with enhanced tumoricidal activity, thereby restoring immune surveillance and delaying tumor progression (116). Additionally, Toll-like receptor (TLR) agonists have demonstrated the capacity to re-educate TAMs, further supporting their therapeutic potential in TNBC (55). Notably, ATM gene deficiency in murine breast cancer cells has been shown to facilitate macrophage repolarization from M2- to M1-like phenotypes within the TME, leading to reduced tumor growth, angiogenesis, and metastatic burden (117). Another axis of interest is the CD47–SIRPα signaling pathway. CD47, a transmembrane protein overexpressed in many malignancies including TNBC, is associated with immune escape and poor prognosis (118). Its interaction with SIRPα, expressed on macrophages, delivers a “don’t eat me” signal that suppresses phagocytosis. This mechanism enables tumor cells to evade immune clearance. Blocking the CD47–SIRPα interaction reactivates macrophage-mediated phagocytosis and enhances anti-tumor responses. Notably, CD47-targeted agents are currently in clinical trials, with encouraging evidence supporting CD47 blockade as an effective strategy for suppressing TNBC development and metastasis (119).

### 4.3 TAM-targeted nanoengineering strategies

Tumor-targeted nanoparticles (NPs) offer a promising platform for precision drug delivery due to their enhanced specificity, penetrability, and biocompatibility, which improve intratumoral drug accumulation while reducing systemic toxicity (120). Haney et al. (121) demonstrated that EVs loaded with paclitaxel or doxorubicin effectively suppressed tumor growth *in vitro* and *in vivo*. In TNBC, where residual cancer stem cells and inflammatory cues persist post-surgery, TAMs are preferentially recruited to tumor margins. Leveraging this, dual-loaded R8-modified liposomes co-encapsulating paclitaxel and resveratrol were developed for macrophage-mediated delivery, effectively suppressing recurrence and downregulating pro-tumor cytokines (122). Additionally, hybrid membranes composed of macrophage and tumor cell membranes were employed to coat DOX-loaded PLGA NPs, markedly enhancing tumor homing and systemic stability, achieving a metastasis-targeting rate of 88.9% (123). Beyond delivery efficiency, TAM-targeted nanoplatforms are increasingly tailored to modulate the tumor immune microenvironment (124). For instance, dextran-coated iron oxide NPs catalyze Fenton-like reactions to generate ROS, promoting TAM polarization toward an M1 phenotype and inhibiting metastasis (125–127). Moreover, MnO-doped DOX nanospheres encapsulated in macrophages enable laser-triggered release at tumor sites, locally decomposing H_2_O_2_ to relieve hypoxia while MnO reduction liberates Mn^2+^ and DOX, amplifying cytotoxicity through enhanced ROS production (128). Together, these studies highlight the transformative potential of TAM-targeted nanoengineering in overcoming drug delivery barriers, modulating immune responses, and improving therapeutic outcomes. Continued innovation in macrophage-based nanotechnology offers promising translational avenues for cancer immunotherapy.

## 5 Conclusion

Tumor-associated macrophages (TAMs) are key orchestrators of triple-negative breast cancer (TNBC) progression, contributing to immunosuppression, angiogenesis, metastasis, and therapeutic resistance. Recent advances highlight various strategies targeting TAMs, including depletion via CSF-1R inhibition, repolarization toward M1-like phenotypes, blockade of the CD47–SIRPα axis, and macrophage-mediated nano-drug delivery. These approaches hold substantial potential to reshape the tumor immune microenvironment and enhance treatment responses.

However, translating TAM targeted therapies into clinical success remains challenging. The functional heterogeneity of TAMs, shaped by ontogeny, spatial localization, and cytokine context, complicates precise targeting. Additionally, the dynamic plasticity of TAM polarization hinders real time monitoring, while the absence of robust biomarkers limits patient stratification and treatment evaluation. Addressing these obstacles will require integrative strategies incorporating single cell technologies, spatial profiling, and biomarker guided trial designs to identify responsive TNBC subgroups. Only through overcoming these translational barriers can TAM directed interventions be effectively implemented to improve outcomes in TNBC patients.
